# Cost-effectiveness analysis of HPV vaccination for the prevention of oropharyngeal cancer in Chinese adolescent males

**DOI:** 10.3389/fpubh.2025.1584956

**Published:** 2025-04-24

**Authors:** Jiajia Li, Ruifeng Li, Ruixi Qin, Hongyun Wang, Di Wang, Liangru Zhou

**Affiliations:** ^1^School of Management, Beijing University of Chinese Medicine, Beijing, China; ^2^School of Population Medicine and Public Health, Chinese Academy of Medical Sciences and Peking Union Medical College, Beijing, China

**Keywords:** HPV vaccine, male adolescents, oropharyngeal cancer, cost-effectiveness analysis, Markov model

## Abstract

**Background:**

In the context of oropharyngeal cancer poised to impose a significant disease burden, this study conducted an economic evaluation of HPV vaccination in Chinese male adolescents for the prevention of HPV-positive oropharyngeal cancer (OPC-HPV+), by constructing a multi-state Markov model from the societal perspective.

**Methods:**

The model estimated the cost, effectiveness, and health utility of the bivalent, quadrivalent, and nonavalent HPV vaccines in preventing OPC-HPV+. Incremental cost-utility ratio (ICUR) was used to evaluate the economic viability of the vaccination strategies. One-way sensitivity analysis and probabilistic sensitivity analysis were employed to assess the model’s stability.

**Results:**

At a vaccine coverage rate of 70%, the incremental cost-effectiveness ratios of the bivalent, quadrivalent, and nonavalent vaccines were all lower than the per capita GDP compared to no vaccination, indicating that the vaccination strategies are highly cost-effective. The nonavalent vaccine has the highest incremental cost-effectiveness ratio, at 64,913.42 yuan ($9,211.86)/QALY. This strategy also has the highest cost, at 112.34 billion yuan, but it provides the best protection outcomes, preventing 2,545,988 cases of persistent HPV infection, 31,186 cases of OPC-HPV+, and 15,138 deaths, saving a total of 2,641,783 QALYs. Sensitivity analysis indicates that the discount rate, vaccine efficacy, HPV infection rate in the general population, and the probability of spontaneous clearance are the main factors affecting the pairwise comparison results of the strategies, which may lead to instability in the cost-effectiveness of the nonavalent vaccine.

**Conclusion:**

HPV vaccination for male adolescents to prevent oropharyngeal cancer is cost-effective compared to no vaccination. China could expand the coverage of the appropriate-priced HPV vaccine to male adolescents in order to reduce the incidence of oropharyngeal cancer, improve male health quality, and protect public health.

## 1 Introduction

Persistent infection with carcinogenic human papillomavirus (HPV) is a significant risk factor for certain malignancies (1). As one of the most common sexually transmitted pathogens globally, both men and women can be infected by HPV (2). An analysis of global cancer incidence due to HPV infection in 2018 showed that HPV caused approximately 620,000 cancer cases in women and 70,000 cancer cases in men (3, 4). Among these, the incidence of HPV-positive oropharyngeal cancer (OPC-HPV+) is showing a significant upward trend (5), with HPV infections accounting for 60–70% of OPC cases in some regions, and the incidence of oropharyngeal cancer is notably higher in men than in women (6–8). Studies predict that OPC-HPV+ will surpass cervical cancer as the most common HPV-related malignancy, and the treatment and ongoing rehabilitation for oropharyngeal cancer are costly, imposing a substantial economic burden on patients (38).

To grow and develop healthily, adolescents need equitable, appropriate, and effective healthcare services, with vaccination being an important means of disease prevention. In 2006, after the launch of the world’s first preventive HPV vaccine, the prevention of HPV-related diseases became a reality. Numerous studies and post-market clinical data have fully confirmed the effectiveness and safety of the HPV vaccine in preventing HPV infection. Vaccination has become an effective strategy to prevent all HPV-related cancers (9). Vaccination strategies vary by country depending on factors such as HPV prevalence, available vaccine types, population structure, healthcare infrastructure, cultural and social factors, and resource availability. According to the World Health Organization’s recommendations, adolescents should receive the HPV vaccine before their first sexual encounter, as the infection rate is still low at this stage, maximizing the vaccine’s effectiveness (10). This recommendation emphasizes the importance of health protection during childhood and adolescence. Timely vaccination not only effectively prevents HPV-related diseases but also lays a solid foundation for the future health of adolescents. Over 50 countries, including the United States, the United Kingdom, Australia, Canada, Germany, and France, have implemented universal HPV vaccination policies (11).

In China, 46% of male oropharyngeal cancers are caused by persistent HPV infection (7). However, China has not included the HPV vaccine in the national immunization program, and only a few provinces have incorporated HPV vaccination into their local public health policies, aiming to promote free vaccination for eligible girls. There is currently no vaccination policy targeting the male adolescents. This study constructs a multi-state Markov model from the societal perspective to conduct a cost-effectiveness analysis of four strategies for preventing oropharyngeal cancer in male adolescents aged 9–14, which includes no vaccination and vaccination with bivalent, quadrivalent, and nonavalent vaccines at varying coverage rates. The results are evaluated using the incremental cost-utility ratio (ICUR) and compared with China’s per capita GDP to conduct an economic evaluation. This approach helps assess the effectiveness and health utility of HPV vaccination for male adolescents in preventing oropharyngeal cancer outside of clinical trials, providing a reference for vaccination strategies.

## 2 Materials and methods

### 2.1 Study design

A Markov model was constructed to assess the cost-effectiveness of HPV vaccination for preventing oropharyngeal cancer in Chinese male adolescents. The model parameters were sourced and quality-controlled through literature review and expert consultations. The model predicted the costs, outcomes, and utilities over a 61-year period for 57.99 million male adolescents aged 9–14 in China in 2023, following the implementation of different vaccination strategies (12). Three vaccination coverage rates were considered: 50, 70%, and the ideal rate of 100%, with two doses of the vaccine administered (10). The candidate strategies were: Strategy 1, vaccinating all individuals in the cohort with the bivalent HPV vaccine; Strategy 2, vaccinating all individuals in the cohort with the quadrivalent HPV vaccine; Strategy 3, vaccinating all individuals in the cohort with the nonavalent HPV vaccine; and the comparison strategy, where no HPV vaccination is administered to any individuals in the cohort.

The study was conducted from societal perspective, considering the resources associated with vaccination, including the cost of vaccination and the treatment cost savings for OPC-HPV+. Since no adverse reaction events have been reported in China’s HPV vaccine safety monitoring, the costs of adverse reactions were not included in this study’s cost estimation. The economic evaluation was expressed using cost-effectiveness, cost-utility, and incremental cost-utility ratio. The willingness-to-pay threshold for each additional QALY was set at the 2023 per capita GDP of China, which is 89,358 yuan (12, 13). Sensitivity analysis was conducted to verify the model’s uncertainty, and the results were presented using tornado diagrams, incremental cost-utility scatter plots, and cost-utility acceptability curves (14).

The model construction and sensitivity analysis were performed using TreeAge Pro. To maintain consistency, the parameters used in the model were adjusted to 2023 using the Consumer Price Index (CPI). A discount rate of 3% was applied to eliminate the effects of time value of money. This study followed the standards set by the 2022 Health Economic Evaluation Report (Consolidated Health Economic Evaluation Reporting Standards 2022; CHEERS 2022) (15).

### 2.2 Research tools and data sources

#### 2.2.1 Markov model and transition probabilities

**Figure 1:**
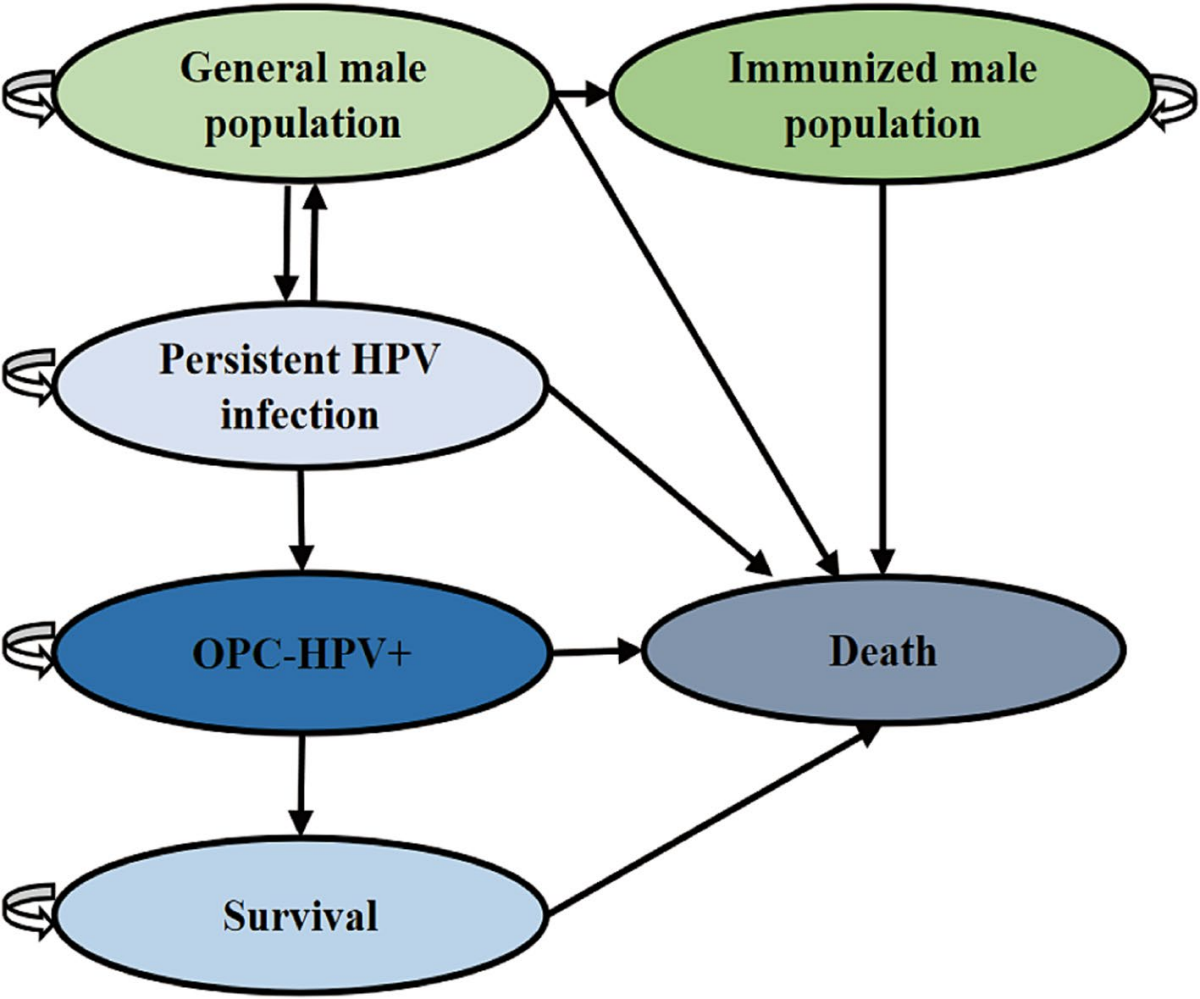
Markov model diagram for vaccination to prevent OPC-HPV+. This model explains the state transitions for OPC-HPV+.

The health states in the Markov model for the study population are divided into: general population, immunized population, HPV infection, HPV-positive oropharyngeal cancer (OPC-HPV+), post-treatment survival of oropharyngeal cancer, and death. These five states were simulated to transition based on the corresponding population transition probabilities (Figure 1 and Table 1). The transition probabilities between states were derived from the results of China’s Seventh National Population Census and publicly published literature (12). Due to the lack of authoritative statistical data on male HPV infection rates and spontaneous clearance rates in China, the two parameters used were derived from global studies and inferences. The model assumed that HPV vaccination only affected whether the general population continues to be infected with HPV. Except for the HPV infection rate and the incidence of oropharyngeal cancer caused by HPV, the transition probabilities for the intervention group and the control group are the same. In the cohort, the “HPV infection” state may progress to the “cancer” state of OPC-HPV+, with transitions occurring according to probabilities into the following three states: If first-line cancer treatment was ineffective, the patient remains in the “cancer” state; If treatment was effective, the patient completed treatment and entered the “survival” state; The patient died from cancer and enters the “death” state. Individuals in the “post-treatment survival” state may relapse into the “cancer” state at the end of each cycle. Based on the assumption of China’s male life expectancy of 74.7 years and the lifelong vaccine efficacy (5, 16), the model was set for 61 cycles.

**Table 1:** Data and sources of Markov model input parameters.

Parameter entry	Numerical value	Reference
The effectiveness of the vaccine in preventing HPV-positive oropharyngeal cancer.		
Bivalent HPV vaccine	73%	Graham DM (21)
Quadrivalent HPV vaccine	83.8%	De Martel (2)
Nonavalent HPV vaccine	89.70%	De Martel (2)
Vaccination dose	2	World Health Organization (10)
Vaccination coverage rates	50, 70, 100%	World Health Organization (10)
HPV infection rates in unvaccinated males	10%	Arbyn M (20)
The probability of physical clears HPV infection automatically	90%	National Health Commission of China (33)
Age-specific all-cause mortality	Dynamic data.	National Bureau of Statistics of China (12)
Incidence of oropharyngeal cancer in men (per 100,000)	3.87	World Health Organization (16)
Proportion of oropharyngeal cancers due to HPV infection	0.46	Lu Y (4)
Case fatality rate of oropharyngeal cancer	0.4	Zhang C (34)
Oropharyngeal cancer survival rate	0.55	Zhang C (34)
Cost of 2 doses of Bivalent HPV vaccine (Yuan)	1,260	China Government Service Procurement Information Platform and Ye Z-H (17, 35)
Cost of 2 doses of quadrivalent HPV vaccine (Yuan)	1,700	China Government Service Procurement Information Platform and Ye Z-H (17, 35)
Cost of 2 doses of nonavalent HPV vaccine (Yuan)	2,760	China Government Service Procurement Information Platform and Ye Z-H (17, 35)
Average treatment cost of oropharyngeal cancer (Yuan)	26,659.32	Song T (39)
The health utility of death	0	Graham DM (21)
Health utility of HPV infection	0.975	Ren Y (36)
Health utility of oropharyngeal cancer	0.528	Qin S (22)
Health utility of oropharyngeal cancer survival	0.769	Graham DM and Habbous S (21, 37)
health utility of the health state	1	Graham DM (21)
Discount rate	0.03	World Health Organization (19)
Period number	61	World Health Organization (16, 23)

#### 2.2.2 Cost parameters

The vaccination costs were derived from the World Health Organization’s position paper on HPV vaccines and publicly published literature in China, which estimate the costs for two doses of imported vaccines and vaccination service fees for different vaccine types (17, 18). The direct treatment costs for oropharyngeal cancer included outpatient services, diagnostics, inpatient treatment, medications, and nursing costs, which were sourced from hospitalization details for cancer patients in the literature (39). Due to a lack of comprehensive data, this analysis did not include the costs of adverse reactions to the vaccine or the indirect costs of oropharyngeal cancer. Based on consensus from Chinese experts, no special treatment was recommended for the initial HPV infection in the general population, so the model set the cost of HPV infection to zero. Based on the recommendations of the World Health Organization, a 3% discount rate was selected for this study (40).

#### 2.2.3 Effectiveness and health utility estimation

Due to the lack of dynamic male HPV transmission data in China, the model did not consider herd immunity effectiveness and instead evaluates the health outcomes of HPV vaccination for preventing oropharyngeal cancer based on overall epidemiological data. The effectiveness was measured by the number of OPC-HPV+ cases prevented by vaccination. Health utility combined both the length of life and the quality of life, adjusted for the quality of life in cancer patients during hospitalization (17). Due to a lack of comprehensive data, this analysis did not include the indirect costs of oropharyngeal cancer. The analysis used quality-adjusted life year (QALY) as the measure. The study assumed an initial health utility value of 1, with the perfect health utility as an age-related variable that decreases with age, applying a 3% discount rate, similar to the cost discounting. Most men during the HPV infection phase and prior to cancer progression were asymptomatic or exhibit mild symptoms, and based on relevant literature from China’s health economics evaluations, this phase was set close to a healthy state with a utility value of 0.975 (8, 20). The cancer utility value for oropharyngeal cancer was derived from a field study in China, where the health utility value was found to be 0.528 (17). The post-treatment survival utility value for oropharyngeal cancer was set to 0.769, based on the empirical research of Graham DM (21, 22).

#### 2.2.4 Vaccine efficacy estimation

Vaccine efficacy was defined as the percentage reduction in disease incidence among vaccinated populations when exposed to the virus. The model assumes that vaccine efficacy does not diminish over time (23, 24). According to the research by Martel et al., the contribution of HPV types 16/18 in head and neck oropharyngeal cancer is 73%, and the contribution of HPV types 6/11/16/18 is 83.8%. The relative contribution of HPV types 6/11/16/18/31/33/45/52/58 is 89.7% (2, 25). The study compares the prevention of HPV infection and subsequent OPC-HPV+ in a cohort of male adolescents who receive bivalent, quadrivalent, and nonavalent HPV vaccination versus those who do not receive the vaccine.

#### 2.2.5 Cost-effectiveness and cost-utility analysis

The model calculated the total costs and the number of OPC-HPV+ cases avoided under different vaccination strategies. A lower number of OPC-HPV+ cases indicated better vaccine effectiveness. Utility is defined as the QALYs saved by reducing oropharyngeal cancer incidence and mortality after vaccination. An incremental cost-utility ratio (ICUR) based on health utility was constructed to report the economic effects of vaccination strategies. The calculation formula is as follows:

$$\frac{\Delta C}{\Delta U}=\frac{C_{I}-C_{0}}{U_{I}-U_{0}}$$

In the formula, ΔC represents the incremental cost; ΔU represents the incremental utility; $C_{I}$ represents the cost of the vaccine intervention group; $C_{0}$ represents the cost of the control group; $U_{I}$ represents the utility of the vaccine intervention group; and $U_{0}$ represents the utility of the control group.

Using China’s per capita GDP in 2023 as a threshold, when the ICUR is less than one times the per capita GDP, the vaccination strategy is considered highly cost-effective. When the ICUR is greater than one but less than three times the per capita GDP, the vaccination strategy is considered cost-effective. When the ICUR exceeds three times the per capita GDP, the vaccination strategy is not considered cost-effective.

#### 2.2.6 Sensitivity analysis

The study performed a one-way sensitivity analysis by setting the numerical fluctuation range for the following parameters: the discount rate fluctuated between 1 and 5%, the HPV infection rate, oropharyngeal cancer incidence, health utility, and treatment costs for oropharyngeal cancer varied by ±20%, and the efficacy values of different types of HPV vaccines fluctuated by ±10%. The tornado diagram visually displays and analyzes the impact of these variations on the model’s predicted results (26). For probabilistic sensitivity analysis, the cost uses a Gamma distribution, and for all transition probabilities and utilities, a Beta distribution (ranging from 0 to 1) is used. A total of 10,000 simulations were conducted to perform cost-utility analysis and calculations. Based on these two types of simulations, scatter plots of the ICUR plane and cost-utility acceptability curves are drawn to visualize the probability that the vaccination strategy is cost-effective at specific willingness-to-pay thresholds.

## 3 Results

### 3.1 Base-case analyses

The model first simulated the results for 57.99 million male adolescents aged 9–14 in China in 2023, implementing three candidate strategies with a 50% coverage rate, along with a comparison strategy—no vaccination (Table 2). With a 3% discount rate, the lifetime cost of oropharyngeal cancer related to HPV for the target population under the no vaccination scenario is 7.72 hundred million yuan. Compared to no vaccination, among the three vaccination strategies, the total cost of administering the nonavalent HPV vaccine is the highest, but it can prevent the most HPV-positive oropharyngeal cancer cases. In terms of vaccination effectiveness, the bivalent HPV vaccine prevented 1,479,990 cases of high-risk HPV persistent infections, 18,128 cases of OPC-HPV+ cases, and 8,800 cases of OPC-HPV+ related deaths; the quadrivalent HPV vaccine prevented 1,698,947 cases of high-risk HPV persistent infections, 20,810 cases of OPC-HPV+ cases, and 10,101 cases of OPC-HPV+ related deaths; the nonavalent HPV vaccine prevented 1,818,563 cases of high-risk HPV persistent infections, 22,275 cases of OPC-HPV+ cases, and 10,813 cases of OPC-HPV+ related deaths.

**Table 2:** Results of disease prevention effectiveness analysis under different vaccination strategies with 50% coverage rate.

Comparison item	Contrast strategy unvaccinated	Strategy 1 bivalent vaccine	Strategy 2 quadrivalent vaccine	Strategy 3 nonavalent vaccine
Reduce persistent high-risk HPV infection (case)	–	1,479,990	1,698,947	1,818,563
Reduce the incidence of OPC-HPV+ (case)	–	18,128	20,810	22,275
Reduce death from OPC-HPV+ (case)	–	8,800	10,101	10,813
Mass of life saved (QALY)	–	1,535,676	1,762,871	1,886,988
Persistent infection with high-risk HPV (case)	4,054,767	2,574,777	2,355,820	2,236,204
Number of OPC-HPV+ (case)	49,666	31,538	28,856	27,391
Death from OPC-HPV+ (case)	24,108	15,309	14,007	13,296
Total cost (Hundred million yuan)	7.72	370.30	517.78	804.65

In the comparison of the results for the three candidate strategies and no vaccination at a 70% coverage rate, the bivalent HPV vaccine prevented 2,071,986 cases of high-risk HPV persistent infections, 25,380 cases of OPC-HPV+ cases, and 12,319 cases of OPC-HPV+ related deaths; the quadrivalent HPV vaccine prevented 2,378,526 cases of high-risk HPV persistent infections, 29,134 cases of OPC-HPV+ cases, and 14,142 cases of OPC-HPV+ related deaths; the nonavalent HPV vaccine prevented 2,545,988 cases of high-risk HPV persistent infections, 31,186 cases of OPC-HPV+ cases, and 14,142 cases of OPC-HPV+ related deaths (Table 3).

**Table 3:** Results of disease prevention effectiveness analysis under different vaccination strategies with 70% coverage rate.

Comparison item	Contrast strategy unvaccinated	Strategy 1 bivalent vaccine	Strategy 2 quadrivalent vaccine	Strategy 3 nonavalent vaccine
Reduce persistent high-risk HPV infection (case)	–	2,071,986	2,378,526	2,545,988
Reduce the incidence of OPC-HPV+ (case)	–	25,380	29,134	31,186
Reduce death from OPC-HPV+ (case)	–	12,319	14,142	15,138
Mass of life saved (QALY)	–	2,149,946	2,468,020	2,641,783
Persistent infection with high-risk HPV (case)	4,054,767	1,982,781	1,676,241	1,508,779
Number of OPC-HPV+ (case)	49,666	24,287	20,532	18,481
Death from OPC-HPV+ (case)	24,108	11,789	9,966	8,971
Total cost (Hundred million yuan)	7.72	515.33	721.80	1,123.41

Under ideal conditions, the model compared the results of three candidate strategies with 100% coverage rate and no vaccination (Table 4). In terms of vaccination effectiveness, the bivalent HPV vaccine prevented 2,919,432 cases of high-risk HPV persistent infections, 35,760 cases of OPC-HPV+ cases, and 17,358 cases of OPC-HPV+ related deaths; the quadrivalent HPV vaccine prevented 3,397,895 cases of high-risk HPV persistent infections, 41,620 cases of OPC-HPV+ cases, and 20,202 cases of OPC-HPV+ related deaths; the nonavalent HPV vaccine prevented 3,637,126 cases of high-risk HPV persistent infections, 44,551 cases of OPC-HPV+ cases, and 21,625 cases of OPC-HPV+ related deaths.

**Table 4:** Results of disease prevention effectiveness analysis under different vaccination strategies with 100% coverage rate.

Comparison item	Contrast strategy unvaccinated	Strategy 1 bivalent vaccine	Strategy 2 quadrivalent vaccine	Strategy 3 nonavalent vaccine
Reduce persistent high-risk HPV infection (case)	–	2,919,432	3,397,895	3,637,126
Reduce the incidence of OPC-HPV+ (case)	–	35,760	41,620	44,551
Reduce death from OPC-HPV+ (case)	–	17,358	20,202	21,625
Mass of life saved (QALY)	–	3,029,278	3,525,743	3,773,975
Persistent infection with high-risk HPV (case)	4,054,767	1,135,335	656,872	417,641
Number of OPC-HPV+ (case)	49,666	13,907	8,064	5,116
Death from OPC-HPV+ (case)	24,108	6,750	3,906	2,483
Total cost (Hundred million yuan)	7.72	732.95	993.81	1,601.57

The above results indicate that several vaccination strategies incur higher costs but provides greater health benefits. The study further used the ICUR value to assess whether the increased costs are economically justifiable, we selected a 70% coverage rate (which aligns with the generally accepted level for vaccine economic evaluations and is close to the coverage standard for vaccine policies in China). Under this scenario, the ICUR values for the three vaccination strategies compared to no vaccination are 23,610.17 yuan ($3,350.52)/QALY, 28,933.26 yuan ($4,105.92)/QALY, and 42,232.58 yuan ($5,993.23)/QALY, all of which are below the 2023 China per capita GDP (89,358 yuan), meaning that compared to no vaccination, the cost of receiving these three vaccines is higher, but the cost per quality-adjusted life year gained does not exceed the threshold for being highly cost-effective, making them acceptable.

After confirming that vaccination is more cost-effective than no vaccination, the incremental cost-utility ratio of the three candidate vaccination strategies were compared pairwise to understand their economic viability. The results show that, compared to the bivalent vaccine, the incremental cost-utility ratio for the quadrivalent vaccine is 64,913.42 yuan ($9,211.86)/QALY, which is less than one times the per capita GDP, thus highly cost-effective; compared to the bivalent vaccine, the incremental cost-utility ratio for the nonavalent vaccine is 123,635.96 yuan ($17,545.17)/QALY, which is greater than one but less than three times the per capita GDP, indicating it is relatively cost-effective; compared to the quadrivalent vaccine, the incremental cost-utility ratio for the nonavalent vaccine is 231,128.07 yuan ($32,799.38)/QALY, which is greater than one but less than three times the per capita GDP, indicating it is relatively cost-effective.

### 3.2 Sensitivity analysis results

**Figure 2:**
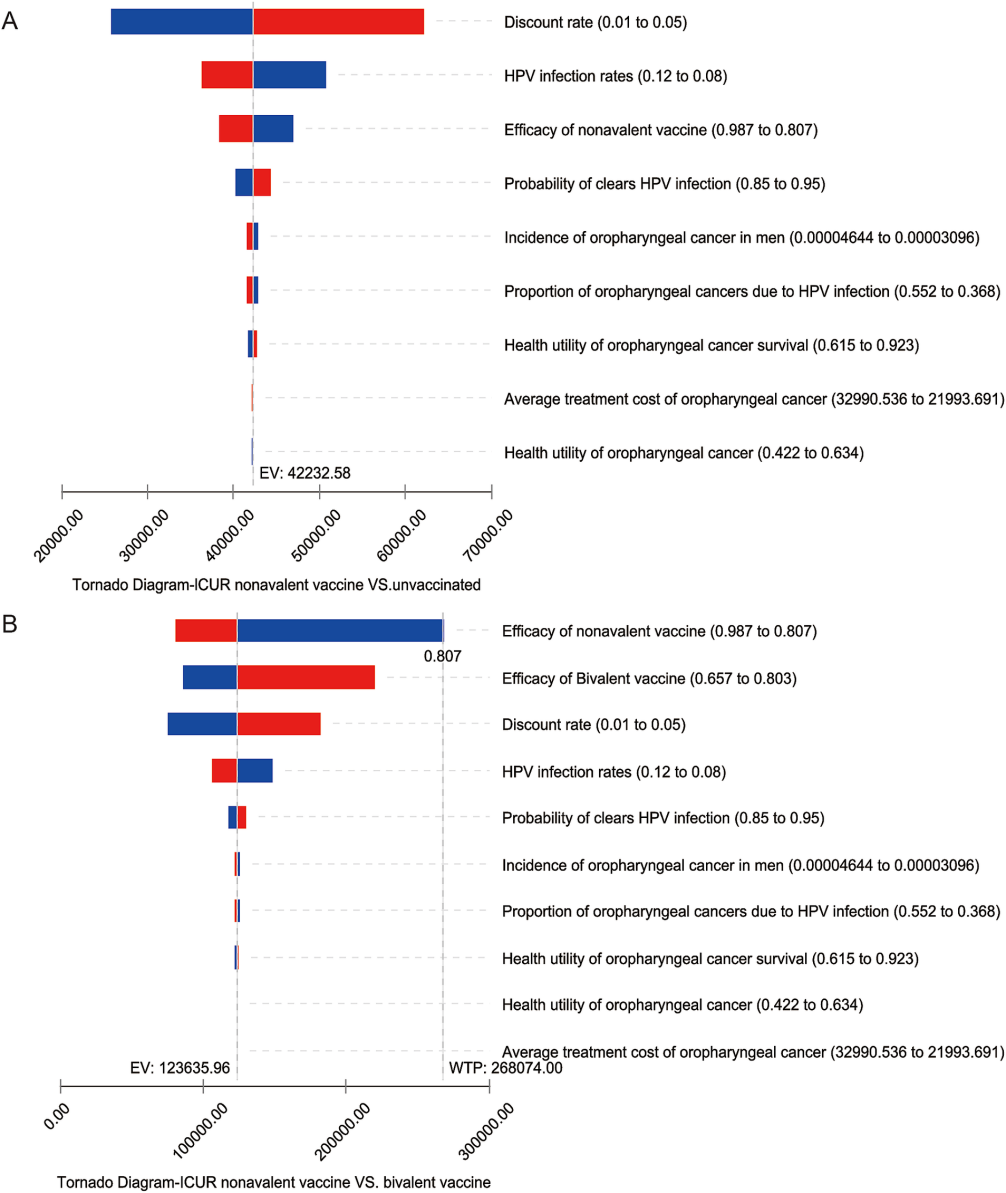
One-way sensitivity analysis tornado diagram. The vertical columns display different factors, while the horizontal range shows the variation in the incremental cost-utility ratio as the factors change. Panel **(A)** shows the change in the ICUR for the nonavalent vaccine compared to no vaccination, panel **(B)** shows the change in the ICUR for the nonavalent vaccine compared to the bivalent vaccine.

The results of the one-way sensitivity analysis indicate that, compared to no vaccination, the discount rate, vaccine efficacy, HPV infection rate in the general population, and the probability of spontaneous clearance are the main factors affecting the baseline results under all three candidate vaccination strategies. However, within the fluctuation ranges, the ICUR does not exceed one times the per capita GDP. The largest fluctuation range is observed in the ICUR comparison between the nonavalent vaccine and no vaccination (Figure 2A). This indicates that, compared to the base case analysis, the incremental cost-effectiveness ratio for all three vaccination strategies remain highly cost-effective, demonstrating robustness.

In the sensitivity analysis comparing the three candidate vaccination strategies pairwise, the discount rate, vaccine efficacy for different vaccine types, HPV infection rate in the general population, and the self-clearance rate are the main factors affecting the baseline results. Some variables cause the incremental cost-utility ratio to exceed the economic threshold. In terms of the discount rate, as the discount rate increases, the incremental cost-utility ratio for the vaccination strategies increases, due to the fact that the cost of vaccination mainly occurs before the model’s time horizon. When the discount rate increases to 0.05 and the HPV infection rate in the general population decreases by 20%, the ICUR of the comparison between the 9-valent and 2-valent vaccines is significantly affected. However, the ICUR remains below three times the per capita GDP of China, which is considered to be relatively cost-effective. The HPV infection rate and self-clearance rate reflect the body’s immune mechanisms. When immunity is weak, the threat of the virus to health is greater, and the health utility brought by vaccination increases. In this case, higher-priced vaccination strategies are more economically viable. Regarding the impact of vaccine efficacy, if the protection efficacy of the nonavalent vaccine against HPV-positive oropharyngeal cancer is reduced by 10%, it would affect the incremental cost-utility ratio for comparing the nonavalent vaccine with the bivalent vaccine, resulting in an ICUR greater than three times China’s per capita GDP, which is considered not cost-effective (Figure 2B). This factor also affects the comparison between the nonavalent and quadrivalent vaccines, as the nonavalent vaccine is significantly more expensive than the bivalent and quadrivalent vaccines. If the protection efficacy of the nonavalent vaccine does not show a significant advantage, the high cost of the nonavalent vaccine will impact its economic viability.

**Figure 3:**
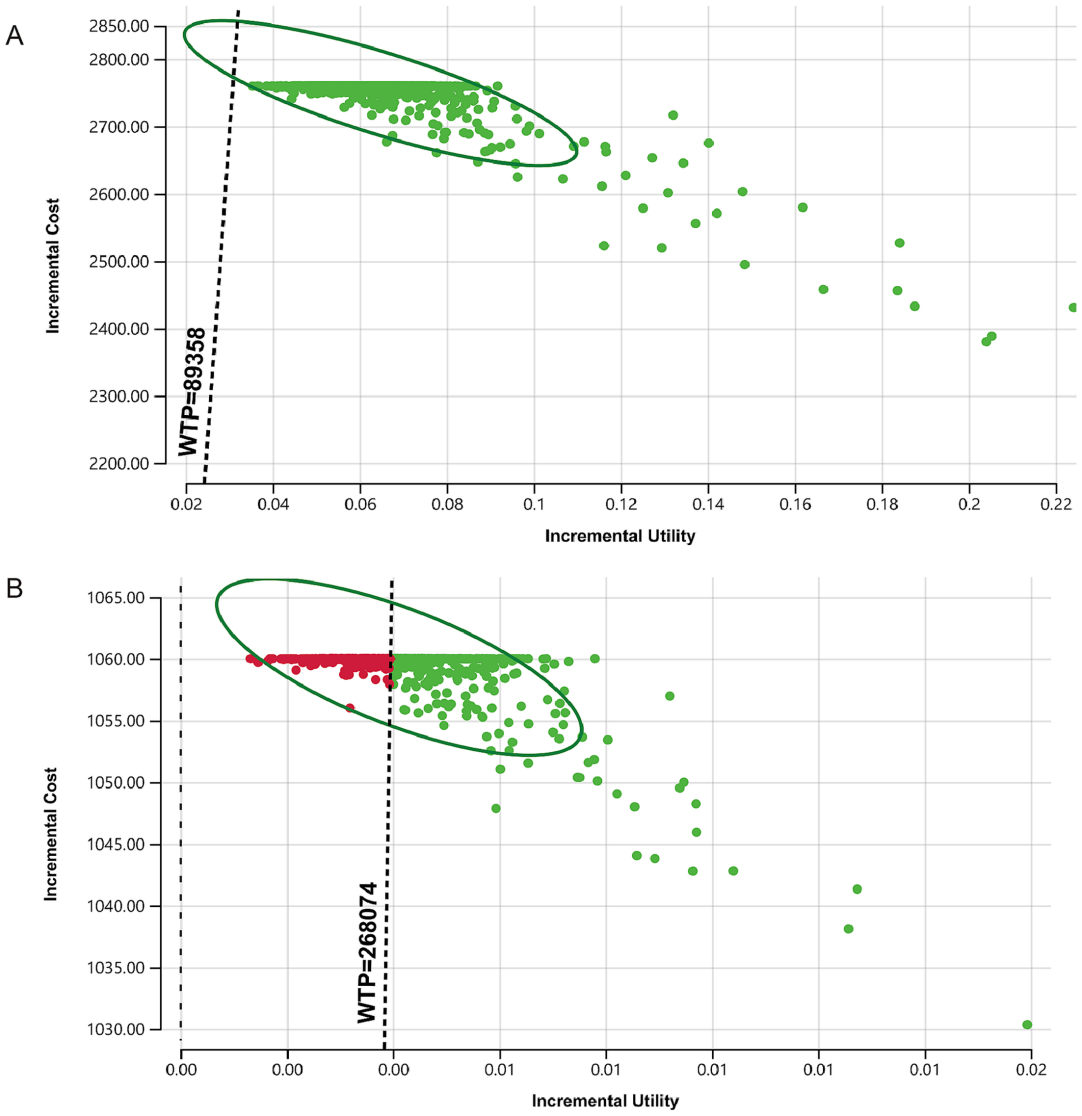
Monte Carlo simulation scatter plots comparing vaccination strategies. The dashed line represents the willingness-to-pay threshold of three times the per capita GDP for economic evaluation. Panel **(A)** shows the change in the incremental cost-utility ratio for the nonavalent vaccine compared to no vaccination, panel **(B)** shows the change in the incremental cost-utility ratio for the nonavalent vaccine compared to the quadrivalent vaccine.

The results of the probabilistic sensitivity analysis show that, compared to no vaccination, the candidate strategies for vaccinating with different HPV vaccine types are cost-effective within a 95% confidence interval. When the willingness-to-pay (WTP) threshold is 89,358 yuan ($12,680.79)/QALY, the probability that the bivalent, quadrivalent, and nonavalent vaccines are cost-effective exceeds 95%. The scatter plot comparing the nine-valent vaccine with no vaccination indicates that the results of the baseline analysis are robust (Figure 3A). Additionally, when comparing the candidate vaccination strategies pairwise, if the WTP threshold is three times China’s per capita GDP, the probability of the quadrivalent and nonavalent vaccines being cost-effective compared to the bivalent vaccine is greater than 95%. The probability of the nonavalent vaccine being cost-effective compared to the quadrivalent vaccine is greater than 50% (Figure 3B). The results of the probabilistic sensitivity analysis are consistent with the one-way sensitivity analysis, both indicating that compared to the quadrivalent vaccine, the economic viability of the nonavalent vaccine is unstable.

**Figure 4:**
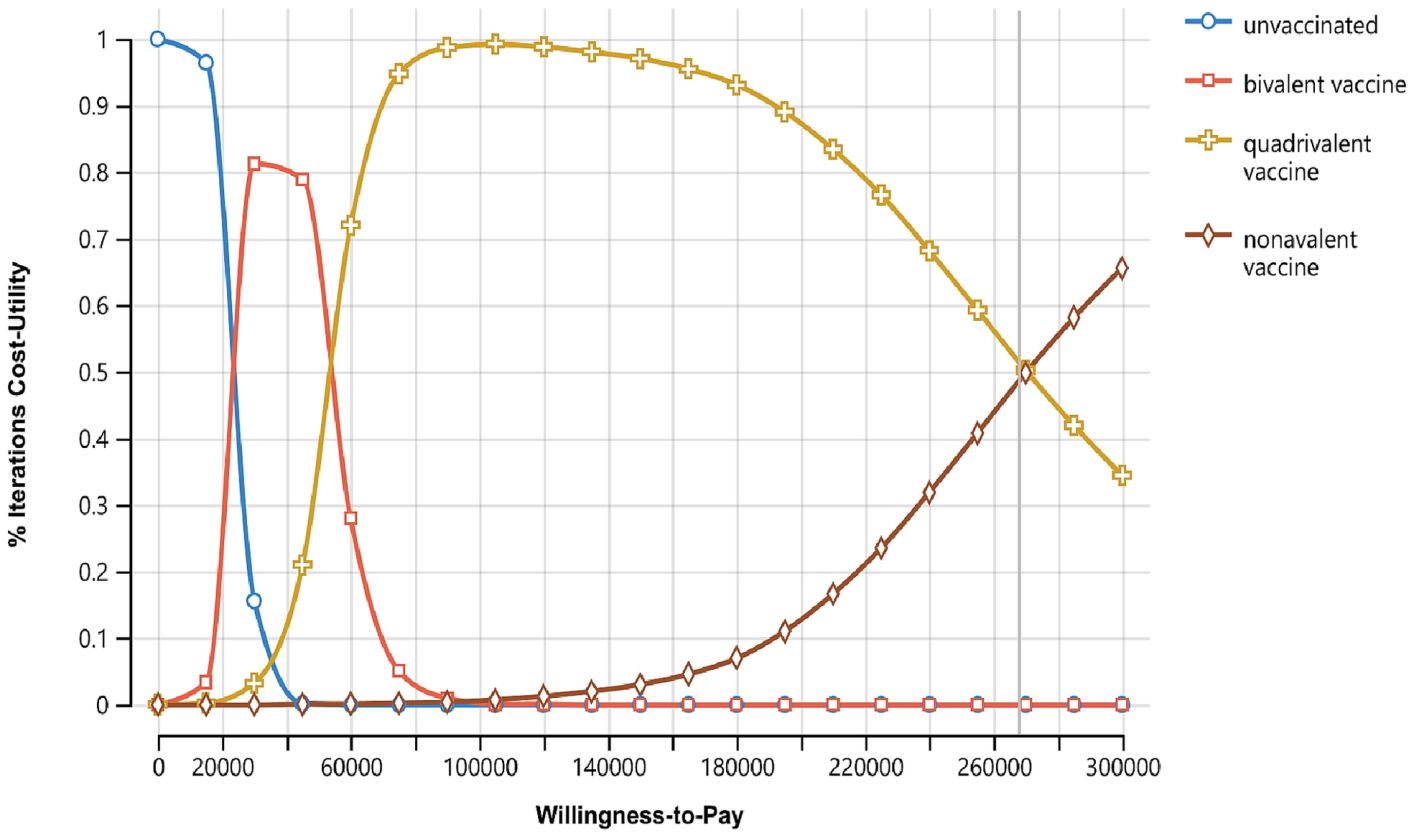
Cost-utility acceptability curves of three vaccination strategies, X-axis represents the willingness-to-pay (WTP) threshold, usually expressed in terms of cost per QALY, Y-axis represents the probability that the intervention is cost-effective at a given WTP threshold.

To further illustrate the selection advantages of various strategies under different willingness-to-pay thresholds, the model generated the acceptability curve for willingness to pay (Figure 4). The sum of the acceptance probabilities for the four strategies is 1, meaning that as WTP increases, the probability of accepting higher-cost strategies increases, while the acceptance probabilities for the other strategies decrease accordingly. The results show that when the WTP for saving one QALY is less than 1 times China’s per capita GDP (89,358 yuan), as WTP increases, the probability of no vaccination decreases, the acceptance probability for the bivalent vaccine first increases and then decreases, while the acceptance probability for the quadrivalent vaccine steadily increases. When the WTP is in the range of 1–3 times China’s per capita GDP, as WTP increases, the likelihood of the quadrivalent vaccine becoming the optimal strategy gradually decreases, while the likelihood of the nonavalent vaccine becoming the optimal strategy gradually increases. This aligns with the general understanding that higher WTP thresholds allow for more expensive interventions to be considered acceptable.

## 4 Discussion

The most direct effect of HPV vaccination is the prevention of HPV-related malignancies. As OPC-HPV+ increases the risk of mortality and morbidity in men, this study focuses the utility of vaccination outcomes on the prevention of OPC-HPV+, estimating the potential benefits of vaccination. The study compares the costs, effects, and health utilities of vaccinating Chinese male adolescents aged 9–14 with the bivalent, quadrivalent, and nonavalent vaccines versus no vaccination, from a societal perspective, to prevent OPC-HPV+.

The results show that, compared to no vaccination, the cost of HPV vaccination is higher, but it improves Chinese male adolescent population’s health utility, with the nonavalent vaccine providing the greatest health benefits. When considering cost-effectiveness, the threshold for a QALY to be considered cost-effective is set at three times China’s per capita GDP in 2023. Under this threshold, the nonavalent vaccine is the most cost-effective strategy compared to no vaccination. In the one-factor sensitivity analysis, the ICURs of the pairwise comparisons of the three vaccination strategies were closely related to factors such as the discount rate, vaccine efficacy, and male HPV infection rate. If the nine-valent vaccine does not provide significantly higher protection against oropharyngeal cancer compared to the four-valent or bivalent vaccines, it could affect the ICUR of the nine-valent vaccine, leading to economic instability. The results of the probabilistic sensitivity analysis further support this possibility. This implies that, under the current economic conditions in China, as well as the context of oropharyngeal cancer epidemiology and associated costs, HPV vaccination is more cost-effective than not vaccinating. However, considering the differences in cost and efficacy among vaccines of varying valency, the choice should be made within the scope of economic feasibility.

The results of this study are consistent with economic evaluations of male HPV vaccination in Hong Kong, Canada, and Netherlands, where the values of incidence rates, cancer treatment costs, and payment thresholds vary due to differences in national economies and cultures (27–29). Studies evaluating the health economics of vaccinating adolescent males, which considered only direct costs, have yielded results consistent with our research, suggesting that HPV vaccination programs are an effective and cost-efficient intervention. The implementation of vaccination programs must address health equity issues arising from income disparities across different regions, particularly concerning healthcare accessibility and vaccine affordability. For instance, providing vaccine price subsidies in economically underdeveloped areas can ensure that low-income populations can afford vaccination.

China has already conducted extensive research on HPV infection in women and the prevention of cervical cancer through HPV vaccination, but there is limited economic research on vaccination for males, and the public is largely unaware of the impact of HPV-related diseases on men. Currently, the development and market supply of high-cost single-dose domestic vaccines are progressing. If domestic vaccines can be expanded to include the male population and significantly reduce vaccination costs, the incremental cost-effectiveness results may greatly improve. The current model only analyzes health utility for oropharyngeal cancer, the most significant HPV-related health issue in men. Due to the lack of data, the costs associated with other low-risk HPV-related conditions in men (such as genital warts, filiform warts, etc.) have not been included, which may lead to the underestimation of the effect of vaccination. If these low-risk HPV-related diseases are considered in the economic analysis, the range of diseases that can be prevented by HPV vaccination will be expanded. HPV vaccination is economically advantageous. Due to the lack of dynamic data on HPV transmission and incidence within the Chinese population, this study has the limitation of not accounting for herd immunity effects. If herd immunity were considered, vaccination at high coverage rates would not only protect vaccinated individuals but also provide significant protection to unvaccinated populations. This would likely increase the number of prevented oropharyngeal cancer cases and persistent HPV infections, thereby enhancing the cost-effectiveness of HPV vaccine (30).

Future research could further analyze and improve the cost-effectiveness of vaccination for Chinese men by establishing dynamic models based on accurate HPV infection data for Chinese male adolescents and considering the herd immunity effect under stable female vaccination coverage and the overall protective impact against other low-risk HPV-related diseases in China (31, 32). Such comprehensive analyses would provide more precise insights into the benefits of extending vaccination programs to include males, thereby informing public health strategies aimed at reducing HPV transmission and associated disease burdens across the population.

## Data Availability

The original contributions presented in the study are included in the article/Supplementary material, further inquiries can be directed to the corresponding author.
