# Supplementary material for: Cost-effectiveness analysis of HPV vaccination for the prevention of oropharyngeal cancer in Chinese adolescent males
Source: Front Public Health. 2025 Apr 24;13:1584956. doi: 10.3389/fpubh.2025.1584956 (PMC12058665; doi:10.3389/fpubh.2025.1584956)
Supplement: Supplementary file 1 [file Data_Sheet_1.docx]

Supplementary Material

## Supplementary Figures


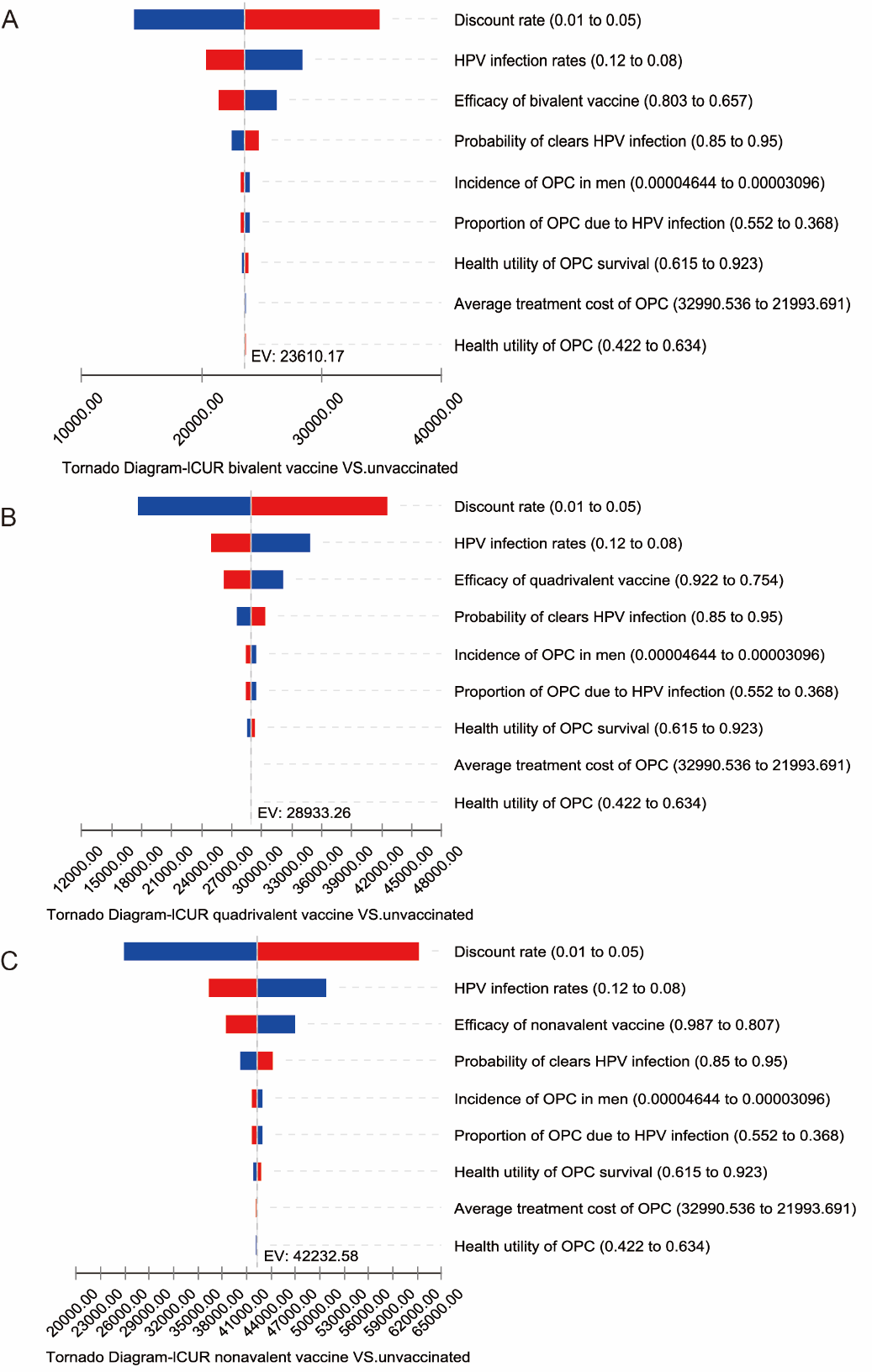


**Figure S1.** One-way sensitivity analysis tornado diagram. The vertical columns display different factors, while the horizontal range shows the variation in the incremental cost-utility ratio (ICUR) as the factors change. Panel (A) shows the change in the ICUR for the bivalent vaccine compared to no vaccination, panel (B) shows the change in the ICUR for the quadrivalent vaccine compared to no vaccination, panel (C) shows the change in the ICUR for the nonavalent vaccine compared to no vaccination.


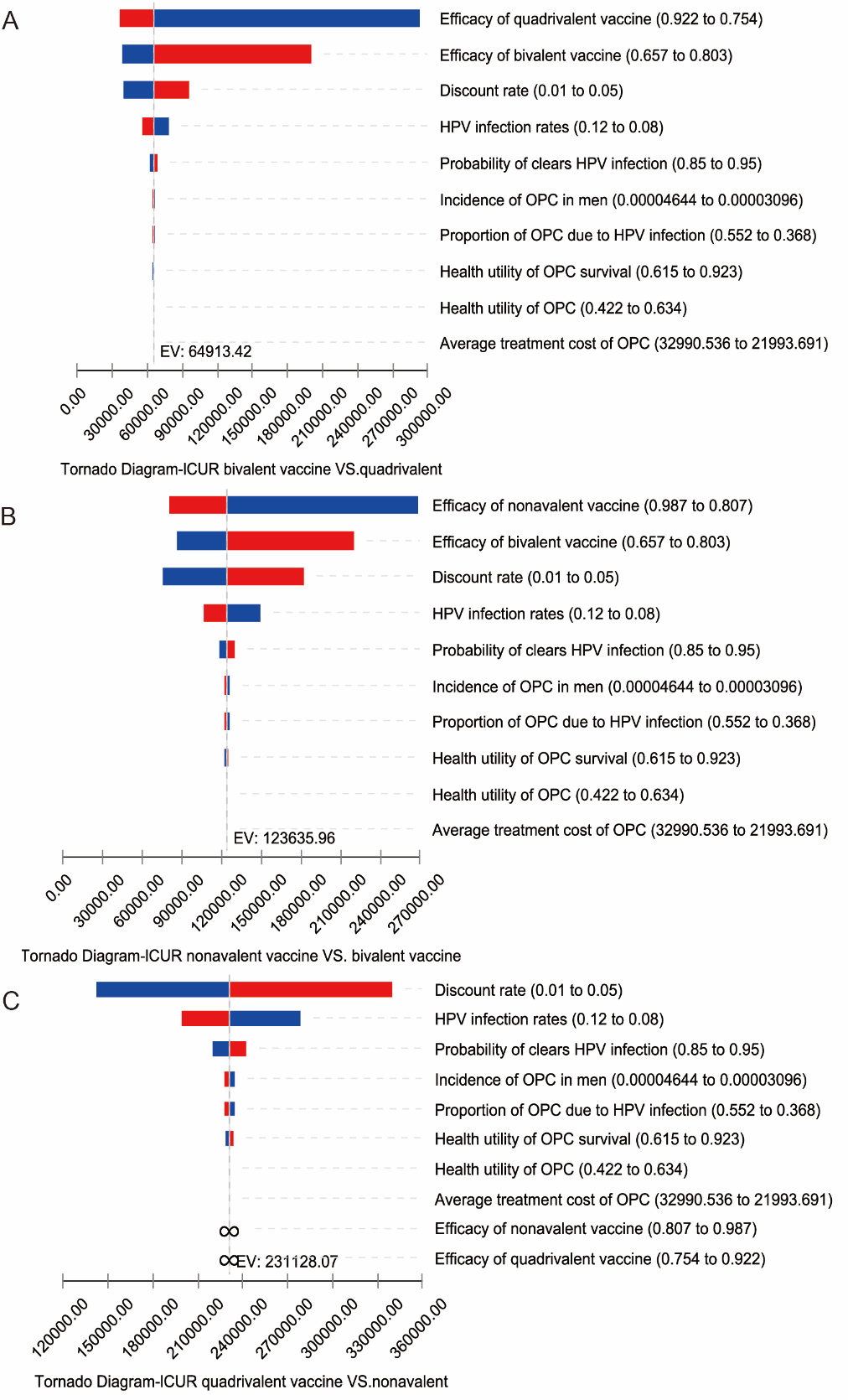


**Figure S2.** One-way sensitivity analysis tornado diagram. The vertical columns display different factors, the horizontal range shows the variation in the incremental cost-utility ratio (ICUR) as the factors change. Panel (A) shows the change in the ICUR for the quadrivalent vaccine compared to the bivalent vaccine, panel (B) shows the change in the ICUR for the nonavalent vaccine compared to the bivalent vaccine, panel (C) shows the change in the ICUR for the nonavalent vaccine compared to the quadrivalent vaccine.
